# Abbreviated versions of the shortened assessment of health literacy for adult emergency department patients: Derivation and testing

**DOI:** 10.1080/2331205X.2021.2024698

**Published:** 2022-04-20

**Authors:** Roland C. Merchant, Sarah J. Marks, Melissa A. Clark, Michael P. Carey, Tao Liu

**Affiliations:** 1 Department of Emergency Medicine, Brigham and Women’s Hospital, Harvard Medical School; 2 Department of Health Services Policy and Practice, School of Public Health, and Department of Obstetrics and Gynecology, Alpert Medical School, Brown University; 3 Centers for Behavioral and Preventive Medicine, the Miriam Hospital, Department of Behavioral and Social Sciences, School of Public Health, and Department of Psychiatry and Human Behavior, Alpert Medical School, Brown University; 4 Department of Biostatistics, Center for Statistical Sciences, Brown University School of Public Health

**Keywords:** Health Communication, Health Education and Promotion, Medicine, Emergency Medicine, emergency medicine, health literacy, adult, mass screening

## Abstract

We aimed to derive and test abbreviated versions of the Shortened Assessment of Health Literacy-Spanish and English (SAHL-S&E) that accurately identify English- or Spanish-speaking lower health literacy adult emergency department (ED) patients. Recursive partitioning of the SAHL-S&E was used to derive four abbreviated versions of the SAHL-S&E by mode of administration (self-administered or staff-administered) and language (English or Spanish). Test performance characteristics of the four abbreviated versions of the SAHL-S&E in distinguishing persons with lower health literacy from those with higher health literacy were assessed against the original full version of the SAHL-S&E. The test performance characteristics of the self-administered English abbreviated SAHL-S&E were: AUC 0.84 (0.79, 0.89), sensitivity 0.84 (0.76, 0.91), and specificity 0.68 (0.61, 0.75); and for the self-administered Spanish version were: AUC 0.88 (0.85, 0.92), sensitivity 0.88 (0.82, 0.93), and specificity 0.78 (0.73, 0.83). For the staff-administered English version, the performance characteristics were: AUC 0.94 (0.91, 0.96), sensitivity 0.98 (0.95, 1.00), and specificity 0.74 (0.69, 0.80), and for the staff-administered Spanish version were AUC 0.89 (0.85, 0.92), sensitivity 0.89 (0.84, 0.94), and specificity 0.80 (0.75, 0.85). Although the four abbreviated versions of the SAHL-S&E performed well they differed by content, length, language and how they are administered, which could add complexity in their routine administration in emergency medicine practice.

## 1. Introduction

Health literacy is defined as the degree to which individuals have the capacity to obtain, process, and understand basic health information and services needed to make health decisions.(Ratzan & R, 2000) Health literacy skills encompass multiple domains, including understanding, interpreting and analyzing health information; applying health information when utilizing healthcare; actively participating in encounters with healthcare workers; navigating the healthcare system; and understanding and providing consent for medical care.(Nielsen-Bohlman & Institute of Medicine (U.S.). Committee on Health Literacy, 2004) Approximately 40% of adult emergency department (ED) patients in the United States (US) have inadequate, lower or marginal health literacy,(Herndon et al., 2011) an estimate signifying that high proportions of US adult ED patients are at risk of receiving healthcare not commensurate with their needs, and consequently suffering from the poor health-related consequences associated with lower health literacy.(Baker et al., 2004; Balakrishnan et al., 2017; Berkman et al., 2011; Cho et al., 2008; Griffey, Kennedy et al., 2014; Sheikh et al., 2016; Smith et al., 2012)

Given the serious problems associated with lower health literacy, accurate, brief and easily administered screening tools are needed to identify adult ED patients with lower health literacy so that ED staff can endeavor to improve the delivery of care to these patients. Multiple instruments measure health literacy, but research on their utility and performance among adult ED patients is in an early stage.(Carpenter et al., 2014; Griffey, Melson et al., 2014; McNaughton et al., 2011; Sarangarm et al., 2017) There is no known optimal instrument for the emergency medicine setting, and none are available that can be self-administered. Common limitations in utilizing current health literacy screening instruments in EDs are their length and complexity, the need for trained staff to administer them, and the requirement for materials and equipment in their administration.

We recently assessed the utility of three short health literacy screening items that do not involve substantial training or equipment and can be administered verbally.(Merchant et al., 2020) However, their performance was suboptimal (receiver operating characteristic (ROC) areas under the curve (AUCs) of 0.58–0.66), as compared to the Shortened Assessment of Health Literacy-Spanish and English (SAHL-S&E),(Lee et al., 2010) and thus we did not recommend the three short health literacy screening items for lower health literacy assessments among adult ED patients. Although using the SAHL-S&E itself would seem to be a logical solution, its routine use in EDs is not practical because it but must be administered by a trained person, and requires materials, equipment and a means of tabulating the results. Self-administration of the SAHL-S&E is not possible because it involves examining the ability to read, pronounce and comprehend 18 health-related words, which makes it challenging to utilize in busy ED settings. An abbreviated instrument derived from the SAHL-S&E that is self-administered instead of staff-administered, and is as accurate as the original would enable expanded screening for lower health literacy in EDs. An abbreviated, modified SAHL-S&E potentially could be self-administered using a mobile device and its results automatically linked to the ED electronic health record (EHR). Incorporation of staff-administered health literacy screening into EHRs has been demonstrated in other healthcare settings.(Cawthon et al., 2014; Sand-Jecklin et al., 2017; Warring et al., 2018)

To address the need for a brief health literacy screening instrument for the emergency medicine setting, in this investigation we aimed to derive and test abbreviated versions of the SAHL-S&E that accurately identify English- or Spanish-speaking adult ED patients with lower health literacy. We used recursive partitioning to create the abbreviated versions, which is a technique helpful in producing screening or testing algorithms resulting in binary categorizations (e.g., lower or higher health literacy).(Blankers et al., 2014; Cai et al., 2019; Yonkers et al., 2010) We specifically were interested in creating abbreviated versions of the SAHL-S&E that could be self-administered, as opposed to staff-administered. However, given that self-administration might not be possible, we also explored developing and testing abbreviated staff-administered versions of the SAHL-S&E.

## 2. Methods

### 2.1. Study design and setting

This investigation involved deriving and then testing abbreviated versions of the SAHL-S&E. Data for the investigation were obtained from a larger study focusing on HIV testing and health literacy among English- or Spanish-speaking adult ED patients.(Merchant et al., 2018) Data from the parent study were divided randomly into a training set (75% of the data) for derivation of the abbreviated SAHL-S&E, and a test set (25% of the data) to evaluate the derived instruments. The parent study involved obtaining health literacy assessments as well as demographic characteristics from adult patients at four urban EDs from 2014 to 2018. Spanish speakers were recruited from an EDs in California and Rhode Island, and English speakers were recruited from an EDs in Alabama and Ohio. The study was approved by each of the hospitals’ institutional review boards.

### 2.2. Study sample

The parent study protocol has been published previously (Merchant et al., 2018) and is summarized here. ED patients were selected at random by bed number using a random number generator for possible inclusion in the study. Research assistants (RAs) reviewed patient EHRs to assess for potential study eligibility, and then confirmed eligibility through an in-person assessment. Eligibility criteria were: age 18–64 years old; English or Spanish speaking; not critically ill, injured or presenting for evaluation of an acute psychiatric illness; not imprisoned or under arrest; and neither intoxicated nor having any other impairment that would preclude informed consent to participate. Due to the nature of the parent study, ED patients were excluded if they had an HIV infection, were not residing in the US, or did not have a contact method for follow up. Because the SAHL-S&E requires an ability to read, ED patients who were unable to read at a 2nd grade level in English or Spanish per the IPT^®^II Spanish or English Oral Test also were excluded. The final study sample comprised 2,749 participants; of these, 1,597 were Spanish speakers and 1,152 were English speakers (Table 1).

### 2.3. Health literacy assessment instrument

We used the SAHL-S&E as the “gold standard” for health literacy determination. In previous research, the SAHL-S&E was correlated *r* = 0.62 with the Spanish version of the Test of Functional Health Literacy in Adults (TOFHLA), correlated *r* = 0.68 with the English version of the TOFHLA,16 and correlated *r* = 0.94 with the English version of the Rapid Estimate of Adult Literacy in Medicine (REALM). The SAHL-S&E contains 18 items (identical items in English and Spanish). During its administration, for each of the 18 items participants are shown a word, asked to pronounce it, and then are shown two words to select from as being most closely associated with the word they pronounced. The two options are a correct key word and an incorrect distractor word. For example, one item tested is “hemorrhoid”, for which the correct key word is “vein” and the incorrect distractor word is “heart”. For a participant to receive a score of one point for a given item, he/she must both pronounce the word correctly and associate it with the correct key word. Scores of ≤14 (out of the 18 items) indicate lower health literacy.

### 2.4. Derivation of abbreviated versions of the SAHL-S&E

Using recursive partitioning with the training data set, we derived eight initial abbreviated English and Spanish versions of the SAHL-S&E (four versions in English, four in Spanish). For these initial versions, we considered items from the original SAHL-S&E for the recursive partitioning models. As an exploration, we also considered years of formal education in the models since education is generally associated with lower health literacy(Gazmararian, Baker et al., 1999; Gazmararian, Parker et al., 1999; Kalichman & Rompa, 2000; McNaughton et al., 2011; Schillinger et al., 2002; Shea et al., 2004; Williams et al., 1995) and its inclusion might improve the models. Each of the eight abbreviated versions of the SAHL-S&E were formed as decision trees (comprised of nodes and branches). The decision trees consisted of items (nodes) from the original SAHL-S&E or years of education that would be assessed according to a specific order (branches). As such, each abbreviated version constituted a series of binary tests designed to distinguish persons with lower health literacy from those with higher health literacy.

Of these initial eight abbreviated versions of the SAHL-S&E, four were derived by language (English and Spanish), and of these four by language, two were self-administered and two required administration by another person (staff-administered). The two self-administered and two staff-administered versions differed by decision tree depth (greater or lesser decision tree depth, i.e., longer or shorter versions). Greater decision tree depth corresponded to more SAHL-S&E items being used to classify someone has having higher vs. lower health literacy. We created the longer and shorter versions in an attempt to determine if shorter versions (to reduce participant burden and time to results) performed as well as longer versions. Creation of decision trees for the self-administered, abbreviated versions relied on the results of the reading comprehension (word association) SAHL-S&E data, whereas the abbreviated staff-administered versions were dependent on both the pronunciation and reading comprehension data. We did not force the abbreviated versions to contain the same items by language or administration. The results from the original, full version of the SAHL-S&E was the standard upon which decision trees for both the self-administered and staff-administered versions were constructed.

We next assessed the test performance characteristics [accuracy, discriminatory abilities (AUC), sensitivity and specificity] of eight initial abbreviated English and Spanish versions of the SAHL-S&E. Accuracy was calculated in the standard fashion as: true positives and negatives/true positives and negatives plus false positives and negatives. The test performance characteristics of the decision trees also were examined in relationship to higher decision tree tuning parameters of cost complexity and minimum split size. We used 10-fold cross-validation of the training dataset with 100 repeats to examine the tuning parameters. This initial assessment provided a preview to the minimum number of items that would be needed for the English and Spanish abbreviated versions of the SAHL-S&E to approximate the test performance characteristics of the full length, original SAHL-S&E. The results of this initial assessment suggested that decision tree depths of ≤8 items had similar test performance characteristics as longer trees, and performance was similar for trees with complexities <0.01 and minimum split sizes > 2 (Supplemental Figures 1–4). This analysis also revealed that the discriminatory abilities of the trees were high (per the AUCs), but sensitivity was lower than specificity.

Based on the results of the initial assessment, we conducted a further exploration of possible decision trees by restricting their depth to eight or fewer, minimum split sizes to < 30 and a cost complexity parameter of 0.0000036. In addition, we considered four loss matrices for the decision trees based on applying the abbreviated SAHL-S&E in clinical practice. The loss matrices accounted for a greater cost of false positives (i.e., misclassifying someone with low health literacy as having high health literacy) than false negatives when using decision trees. The loss matrices corresponded to the same, double, triple or quadruple the penalty for misclassifying an individual with low health literacy as having high health literacy than misclassifying an individual with high health literacy as having low health literacy. We assessed the test performance characteristics of the possible decision trees by their depths and penalties (Supplemental Figure 5). This evaluation suggested which decision trees had similar test performance characteristics for the shorter tree depths according to their respective penalties. Shorter decision trees were selected based on a minimum tree depth and penalty to achieve a minimum sensitivity of 0.80 and specificity of 0.80. Longer trees were selected based on minimum tree depths when the AUC plateaued at correspondingly high sensitivity levels. This work resulted in eight initial abbreviated versions of the SAHL-S&E (four in English, four in Spanish) that varied by administration (self-administered or self-administered) and by length (longer and shorter).

### 2.5. Testing of the final versions of the abbreviated SAHL-S&E

From our review of the eight initial abbreviated versions of the SAHL-S&E and their composition, we noted that the longer versions were only 2–3 items greater than the shorter versions, yet the longer versions had better test performance characteristics. In addition, we noted that not all trees included years of education in its decision tree (only the English staff-administered, longer tree), and its inclusion had minimal effects on performance. Based on these observations, we decided to consider further only the longer trees and removed years of education from all trees. This action resulted in four final abbreviated versions of the SAHL-S&E: two in English (self-administered and staff-administered) and two in Spanish (self-administered and staff-administered). The test performance characteristics (AUC, accuracy, sensitivity, specificity, predictive value and likelihood ratios) of the final four abbreviated versions of the SAHL-S&E were assessed using the test dataset. For all metrics, bootstrapping with replacement from the test data set was used to quantify stability of the model and range of performance using 95% confidence intervals (CIs) based on 10,000 replicates.

## 3. Results

### 3.1. Initial eight abbreviated versions of the SAHL-S&E and their test performance characteristics

**Figure 1.**
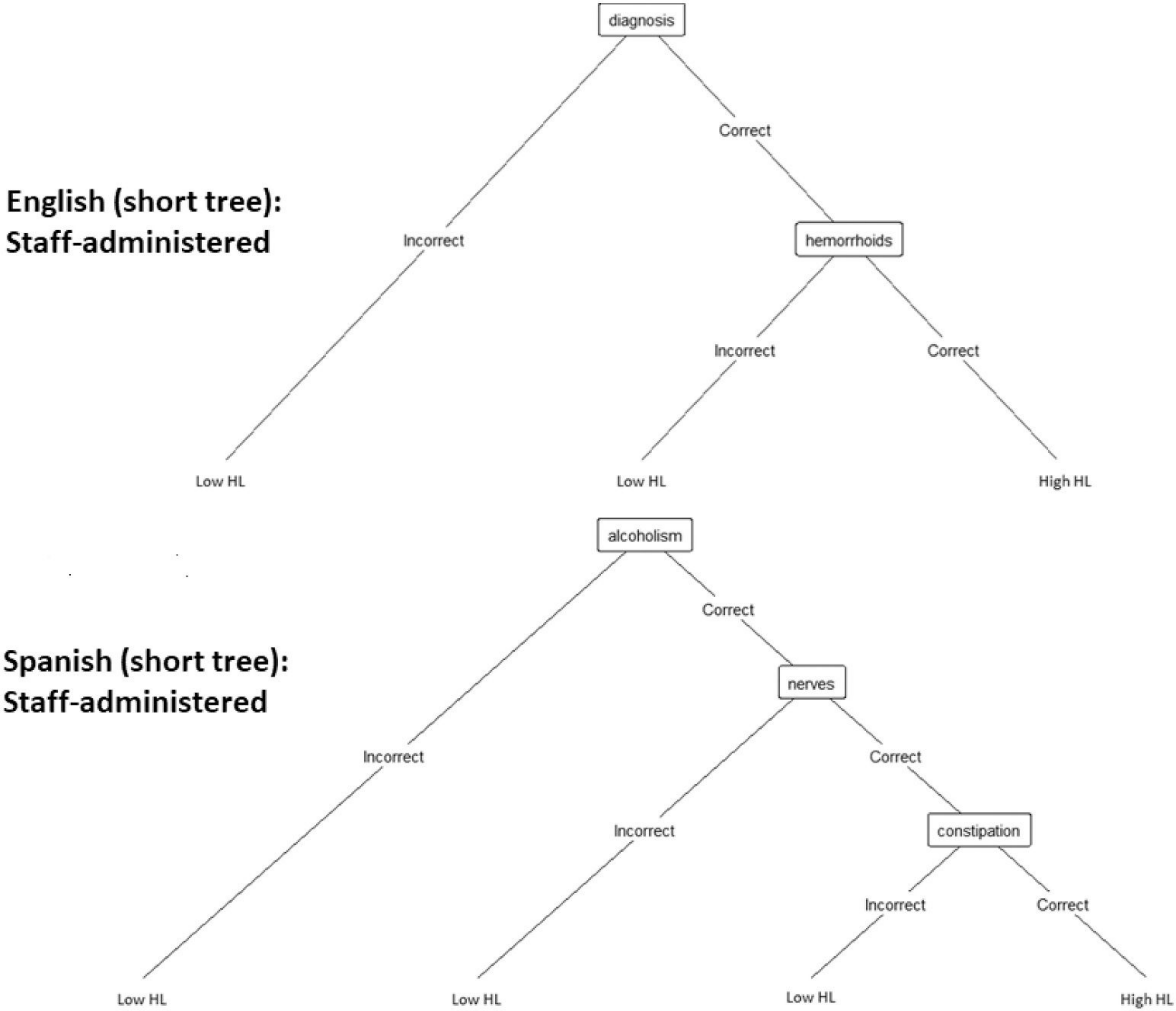

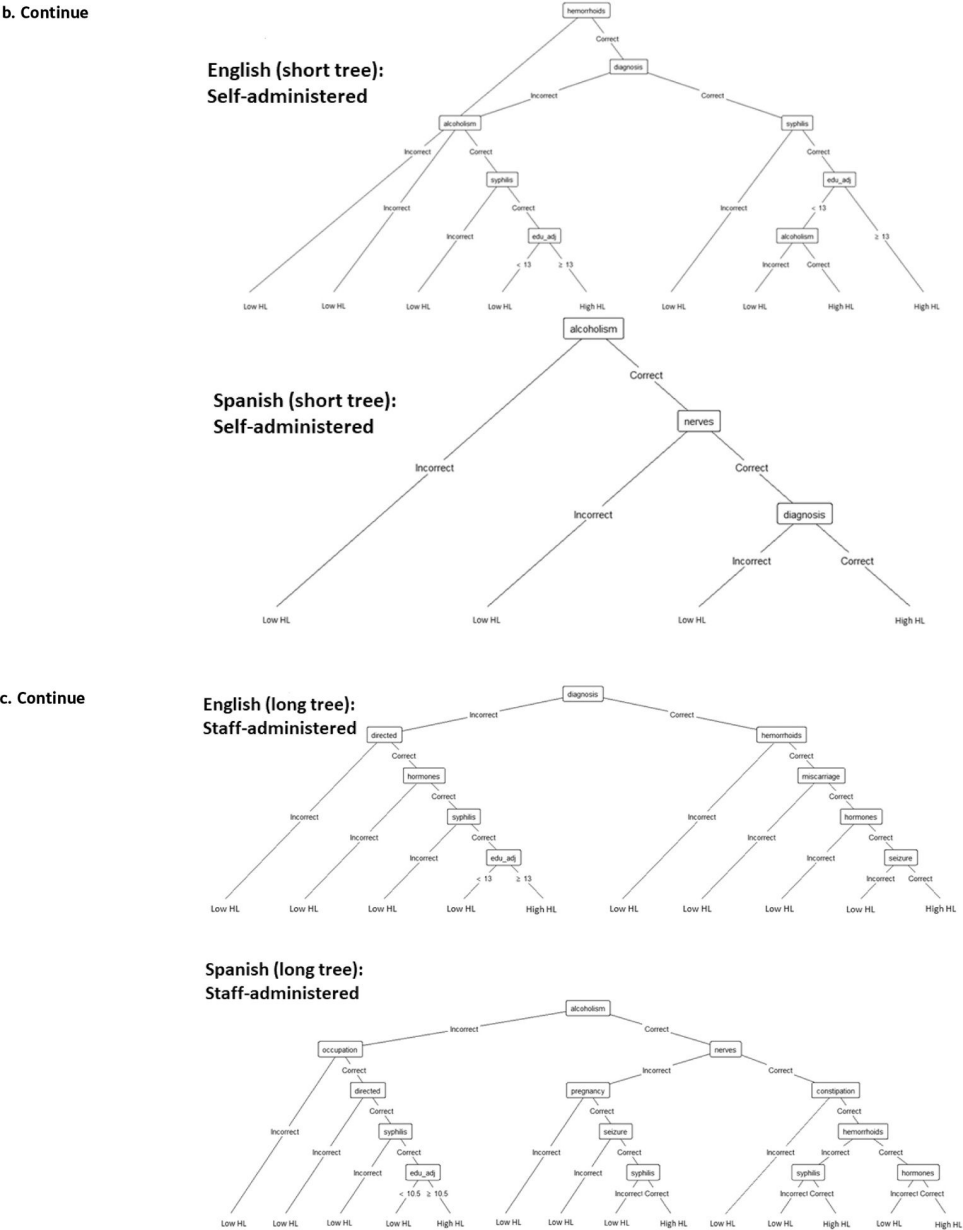

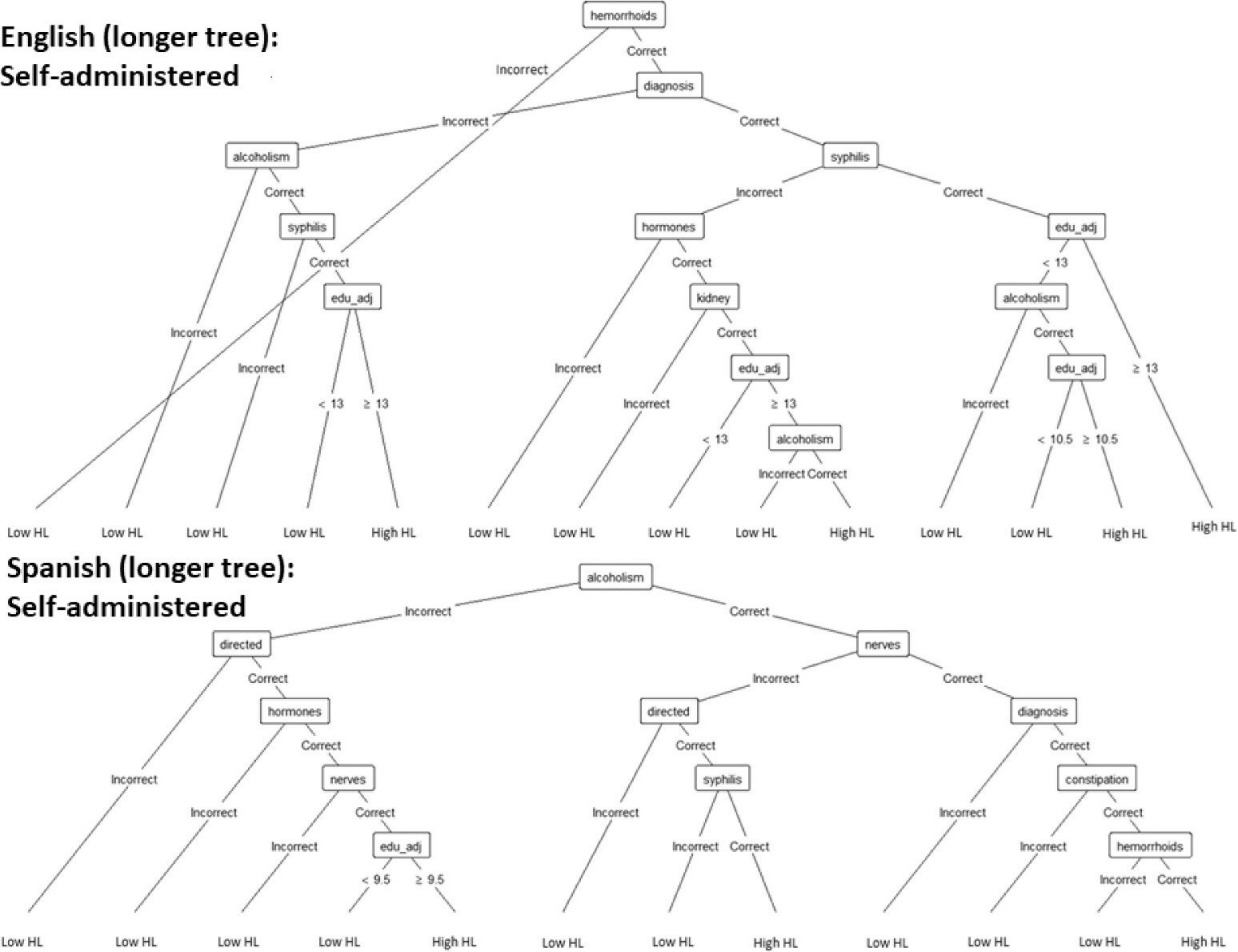
Decision trees derived from the training dataset representing the eight abbreviated SAHL-S&E versions by language, length and administration mode.

The eight decision trees derived from the training dataset representing the eight abbreviated SAHL-S&E versions by administration mode (self-administered or staff-administered), length (shorter or longer) and language (English or Spanish) are provided in Figure 1. The nodes on the trees indicate items from the SAHL-S&E selected during recursive partitioning for each version. Tree length was greatest for the English self-administered versions, as compared to the English staff-administered versions and the Spanish self- and staff-administered versions. The test performance characteristics of the decision trees in identifying participants with lower health literacy using the training dataset are provided in Table 2. Discrimination of lower vs. higher health literacy (per AUCs) was high and tended to be better for longer decision trees. Accuracy and sensitivity also trended higher for longer decision trees. Test performance characteristics for the Spanish self-administered and staff-administered versions were similar, but were slightly lower for the English self-administered than the staff-administered versions.

### 3.2. Test performance characteristics of the final four abbreviated versions of the SAHL-S&E

Table 3 provides the test performance characteristics using the test dataset for the final four decision trees (Figure 1) representing abbreviated versions of the SAHL-S&E by administration mode (self-administered or staff-administered) and language (English or Spanish). Discrimination between lower and higher health literacy remained high (AUCs 0.88 to 0.94). Negative predictive values and negative likelihood ratios were higher than positive predictive values and positive likelihood ratios, and sensitivity was greater than specificity for the four versions. For English, the staff-administered versions performed better than the self-administered versions, while for Spanish the performance of the self- and staff-administered versions were similar.

## 4. Discussion

In this investigation, we derived and then tested abbreviated versions of the SAHL-S&E intended to substitute for the original, full version of the SAHL-S&E. Our hope for this work was to present a shortened and self-administered instrument that rapidly and accurately identified ED patients with lower health literacy. However, we discovered differences in the test performance characteristics of the staff-administered and self-administered versions that precluded our presentation of a single abbreviated screening instrument. As such, the products of our investigation instead are a set of four screening instruments in English and Spanish, two of which are staff-administered, while two can be self-administered. We did achieve our goal to produce instruments that are markedly shorter than the original SAHL-S&E and have high discriminatory abilities (AUCs) so that they can accurately distinguish lower from higher health literacy. The instruments notably have high negative predictive values, which indicates that if a patient is classified as not having lower health literacy, then there is confidence that this classification is correct. In other words, a negative screening test using these instruments helps to “rule out” lower health literacy. On the other hand, the instruments ability to identify those with lower health literacy (i.e., “rule in”, as shown by the positive predictive values) is only moderate. Of course, predictive value is affected by the prevalence of the population in which the screening test will be administered. The likelihood ratios, which are not so affected, also suggest stronger ability to “rule out” than “rule in” lower health literacy using these instruments.

The difference in performance of the staff-administered and self-administered abbreviation versions of the SAHL-S&E according to language bears specific attention, and likely will affect how they can be used in the ED setting. Of note, the staff-administered abbreviated English version had greater test performance characteristics than the self-administered version, while the test performance characteristics of the Spanish staff- and self-administered abbreviated versions were similar. A possible explanation for this difference by language could be related to what the SAHL-S&E measures (ability to read by pronouncing words, and ability to comprehend by matching words to similar other words) and the distinction between the English and Spanish languages. Unlike English, Spanish is a phonetic language, which could make the pronunciation component of the SAHL-S&E for Spanish less important. That is, because pronunciation is easier in Spanish, inability to read and pronounce words could be a lesser indicator of health literacy. In practice, this finding implies that for English speakers, the staff-administered version would be the preferred screening test for health literacy, but either the staff- or self-administered versions would be appropriate for Spanish speakers. However, to identify lower health literacy patients, this difference by language would require greater effort in time and labor for English speakers by ED staff than for Spanish speakers. Although not investigated in this study, voice recognition software might in the future be helpful to overcome this difference in administration.

A valid concern about the four different abbreviated versions is whether they measure the same constructs of health literacy, particularly in comparison to the original full version of the SAHL-S&E. It is possible that although the abbreviated instruments perform well in regard to test performance characteristics to the original full version, this type of assessment ignores any differences in what constructs of health literacy they actually assess. The self-administered versions focus on comprehension and assume the more general literacy skills of word pronunciation, whereas the staff-administered versions assess both. In turn, the greater reliance on comprehension for the Spanish-language versions suggest it also assumes greater general literacy skills than the English version. The constructs of health literacy are measured by the abbreviated versions are limited by what the original full version of the SAHL-S&E itself measures, and as such likely do not measure other constructs. The intended purpose for creating the abbreviated versions is so that they can be used as substitutes for more comprehensive assessments of health literacy. One consideration of their practical usage is that they might serve as a screening tool for further health literacy evaluation. For example, the self-administered versions could be used as an initial screening test that indicates which patients might need further evaluation of their health literacy. Related to the concern about differences in what the four abbreviated versions actually measure is that the discrimination thresholds selected for the abbreviated versions reflect a binary categorization of health literacy, which also reflects that of the categorization employed by the original full version of the SAHL-S&E. This categorization likely oversimplifies health literacy skills. Future research should investigate the constructs the abbreviated versions might actually measure, the binary categorization of health literacy they assess, as well as their possible usage as initial screening tools.

We acknowledge several study limitations. First, the abbreviated versions were derived from, and tested against, the original full version of the SAHL-S&E, as opposed to another health literacy assessment instrument. As such, we do not know how well the abbreviated versions perform against other instruments. The abbreviated versions should be evaluated against other instruments in future research. Second, the determination of health literacy in this study depends on the accuracy of the SAHL-S&E. As shown in other studies, health literacy screening instruments when applied in the same ED patient population have rendered different conclusions about patients’ health literacy skills.(Carpenter et al., 2014) Third, our objective was to maximize detection of persons with lower health literacy, which greatly influenced the resultant composition of the instrument. As such, the abbreviated versions of the SAHL-S&E we produced are intended to identify lower health literacy individuals and to tolerate potential misclassification of those with higher health literacy as opposed to misclassification of those with lower health literacy. This objective and the resultant versions affect not only their performance but their use in clinical practice. Fourth, although testing of the abbreviated versions of the SAHL-S&E performance was conducted using a dataset separate than used to derive them, the underlying population is the same for both datasets. Their performance might differ when examined in EDs with dissimilar demographic characteristics than reflected in the four EDs included in our study. Future research should examine how well these abbreviated versions of the SAHL-S&E perform in other EDs. Furthermore, additional assessments (e.g., test-retest reliability, other psychometric assessments) will need to be performed to assess these instruments. Fifth, the difference in items in the abbreviated versions make them challenging to compare by language. Sixth, the use of recursive partitioning likely resulted in versions of the SAHL-S&E that suggest binary categorizations of health literacy. Other methodologies could have produced different versions. Seventh, this study involved deriving and assessing the abbreviated instruments, but not evaluating their utility in clinical or research practice. This evaluation should be conducted in future research.

In conclusion, we produced abbreviated versions of the SAHL-S&E for English and Spanish speakers that can be used to identify lower health literacy adult ED patients. Self-administration is feasible for the abbreviated SAHL-S&E with minor differences in accuracy when used with English vs. Spanish speakers. Research using the abbreviated instruments should assess their feasibility, acceptability, and accuracy across multiple ED settings.

## Supplementary Material

Supplementary Figures

Supplemental data for this article can be accessed here

## Figures and Tables

**Table 1.** Demographic characteristics of study participants

	Spanish speakers (*n =* 1597)		English speakers (*n =* 1152)	
	n	%	n	%
Gender				
Male	607	38.0	467	40.5
Female	990	62.0	685	59.5
Age in years (median (IQR))	46	(37–54)	42	(30–52)
Current marital status				
Married	716	44.8	230	20.0
Domestic partner	150	9.3	10	0.9
Divorced	156	9.8	193	16.8
Widowed	40	2.5	35	3.0
Separated	116	7.3	57	5.0
Never married	337	21.1	559	48.5
Unmarried	82	5.1	68	5.9
Race				
White	1206	75.5	345	30.0
Black	388	24.3	776	67.4
Other	3	0.2	31	2.7
Health insurance^1^				
Private	92	5.8	271	23.5
Governmental	832	52.1	580	50.4
Private and governmental	6	0.4	40	3.5
None	662	41.5	258	22.4
Health literacy (per SAHL-S&E)				
Lower	1041	65.2	736	63.9
Higher	556	34.8	416	36.1
Years of formal education^2^				
Fourth grade or less	160	10.0	0	0.0
Fifth to eighth grade	450	28.2	20	1.7
Some high school	344	21.5	199	17.3
Completed high school or GED/HSE	427	26.7	453	39.3
Completed some college coursework	138	8.6	346	30.0
Completed college degree/graduate degree	71	4.5	134	11.6

IQR: interquartile range; SAHL-S&E: Short assessment of health literacy-Spanish and English; GED/HSE: general educational development/high school equivalency

**Table 2.** Test performance characteristics for the initial eight decision trees identifying lower health literacy (training data set)

Instrument version	Tree depth	Penalty	AUC	Accuracy	Sensitivity	Specificity
English: Non self-administered	2	2	0.88	0.84	0.92	0.80
English: Non self-administered	5	2	0.93	0.86	0.86	0.86
English: Self-administered	5	3	0.80	0.73	0.75	0.73
English: Self-administered	7	3	0.83	0.72	0.80	0.68
Spanish: Non self-administered	3	4	0.83	0.79	0.84	0.77
Spanish: Non self-administered	5	4	0.89	0.81	0.90	0.76
Spanish: Self-administered	3	4	0.81	0.76	0.79	0.74
Spanish: Self-administered	5	3	0.87	0.81	0.87	0.78

AUC: area under the curve

**Table 3.** Test performance characteristics for the final four decision trees identifying lower health literacy (test data set)

	Tree depth	Penalty	AUC 95% CI	Accuracy 95% CI	Sensitivity 95% CI	Specificity 95% CI	PPV 95% CI	NPV 95% CI	PLR 95% CI	NLR 95% CI
English: Staff-Administered	4	2	0.95 (0.92, 0.98)	0.90 (0.87, 0.93)	0.91 (0.85, 0.94)	0.90 (0.85, 0.94)	0.83 (0.76, 0.90)	0.94 (0.91, 0.98)	8.9 (6.0, 14.8)	0.10 (0.04, 0.16)
English: Self-Administered	6	3	0.87 (0.83, 0.91)	0.81 (0.76, 0.85)	0.80 (0.72, 0.87)	0.82 (0.76, 0.87)	0.71 (0.63, 0.79)	0.88 (0.83, 0.92)	4.3 (3.2, 6.2)	0.25 (0.15, 0.35)
Spanish: Staff-Administered	5	4	0.96 (0.93, 0.97)	0.86 (0.82, 0.90)	0.93 (0.88, 0.98)	0.82 (0.76, 0.87)	0.74 (0.66, 0.81)	0.96 (0.92, 0.99)	5.0 (3.8, 7.2)	0.08 (0.03, 0.15)
Spanish: Self-Administered	5	3	0.85 (0.81, 0.89)	0.83 (0.79, 0.86)	0.86 (0.80, 0.91)	0.81 (0.76, 0.86)	0.70 (0.64, 0.77)	0.91 (0.87, 0.95)	4.5 (3.5, 6.0)	0.18 (0.11, 0.25)

AUC: area under the curve

PPV: positive predictive value

NPV: negative predictive value

PLR: positive likelihood ratio

NLR: negative likelihood ratio

CI: confidence interval

## PUBLIC INTEREST STATEMENT

People with lower health literacy are at higher risk of receiving healthcare that is not adequate for their needs, and consequently suffering poor health-related outcomes. An estimated 40% of adult emergency department patients in the United States have lower health literacy, but health literacy screening is not common in this setting. In this investigation, we sought to derive for English- or Spanish-speaking adult emergency department patients abbreviated health screening instruments from an established longer health screening tool, the Shortened Assessment for Health Literacy (SAHL-S&E). Our goal was to derive abbreviated instruments that ideally could be self-administered. Through a multi-step process, we produced four abbreviated versions of the SAHL-S&E that performed well, but differed by content, length, language and how they are administered (self- or staff-administered). Future research will be needed to determine what role they might have in their routine use for health literacy screening in emergency medicine practice.

## Footnotes

1. 8 people missing data on health insurance.

2. 7 people missing data on health insurance.
