# Supplementary material for: Abbreviated versions of the shortened assessment of health literacy for adult emergency department patients: Derivation and testing
Source: Cogent Public Health. Author manuscript; Available in PMC 2026 Apr 29. (PMC13122187; doi:10.1080/2331205X.2021.2024698)
Supplement: Supplementary Figures [file NIHMS1769169-supplement-Supplementary_Figures.docx]

Supplemental Figure 1: Mean metrics for initial grid search by cost complexity, tree depth, and minimum split size for English staff-administered version


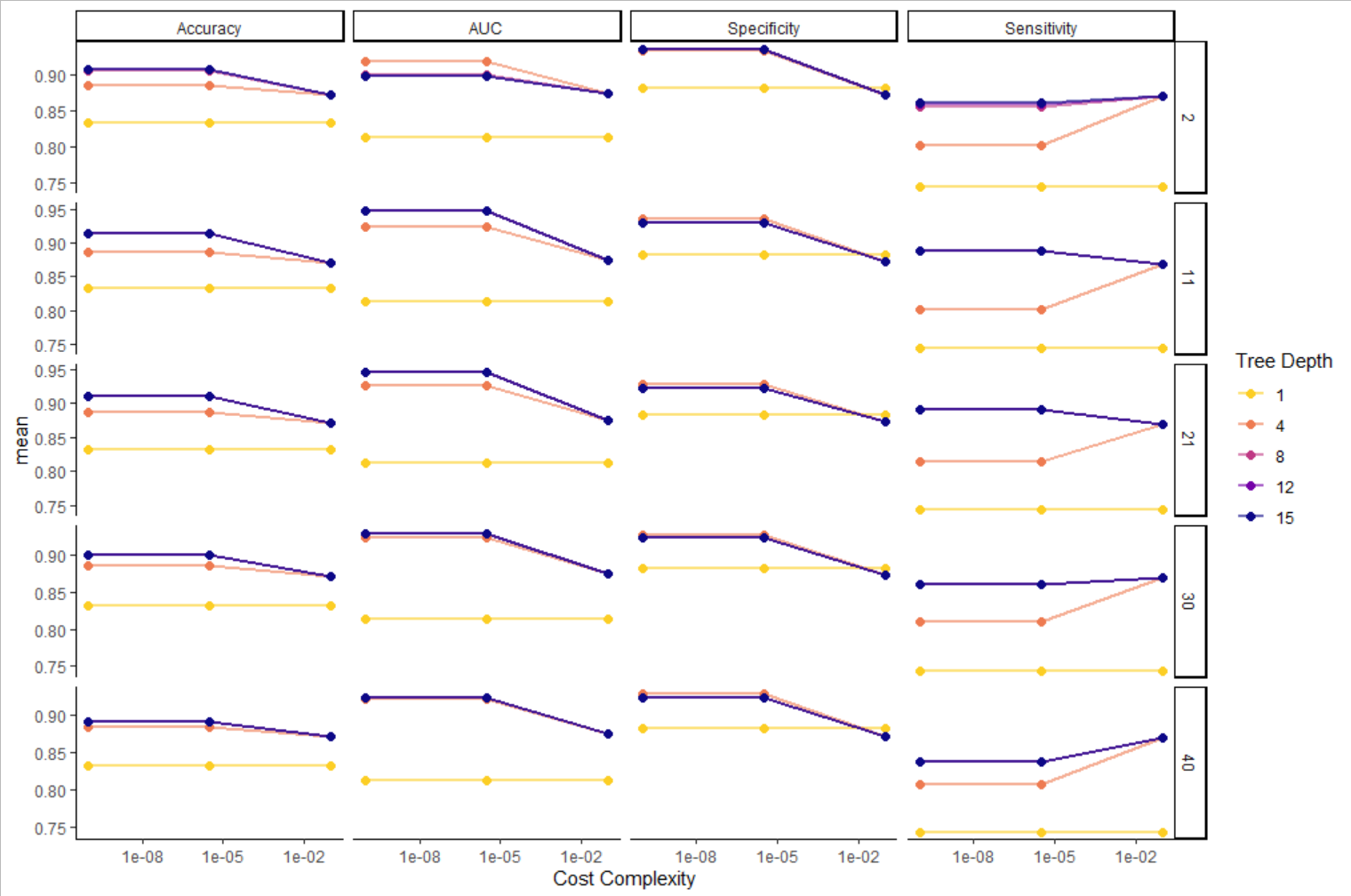


Supplemental Figure 2: Mean metrics for initial grid search by cost complexity, tree depth, and minimum split size for Spanish staff-administered version


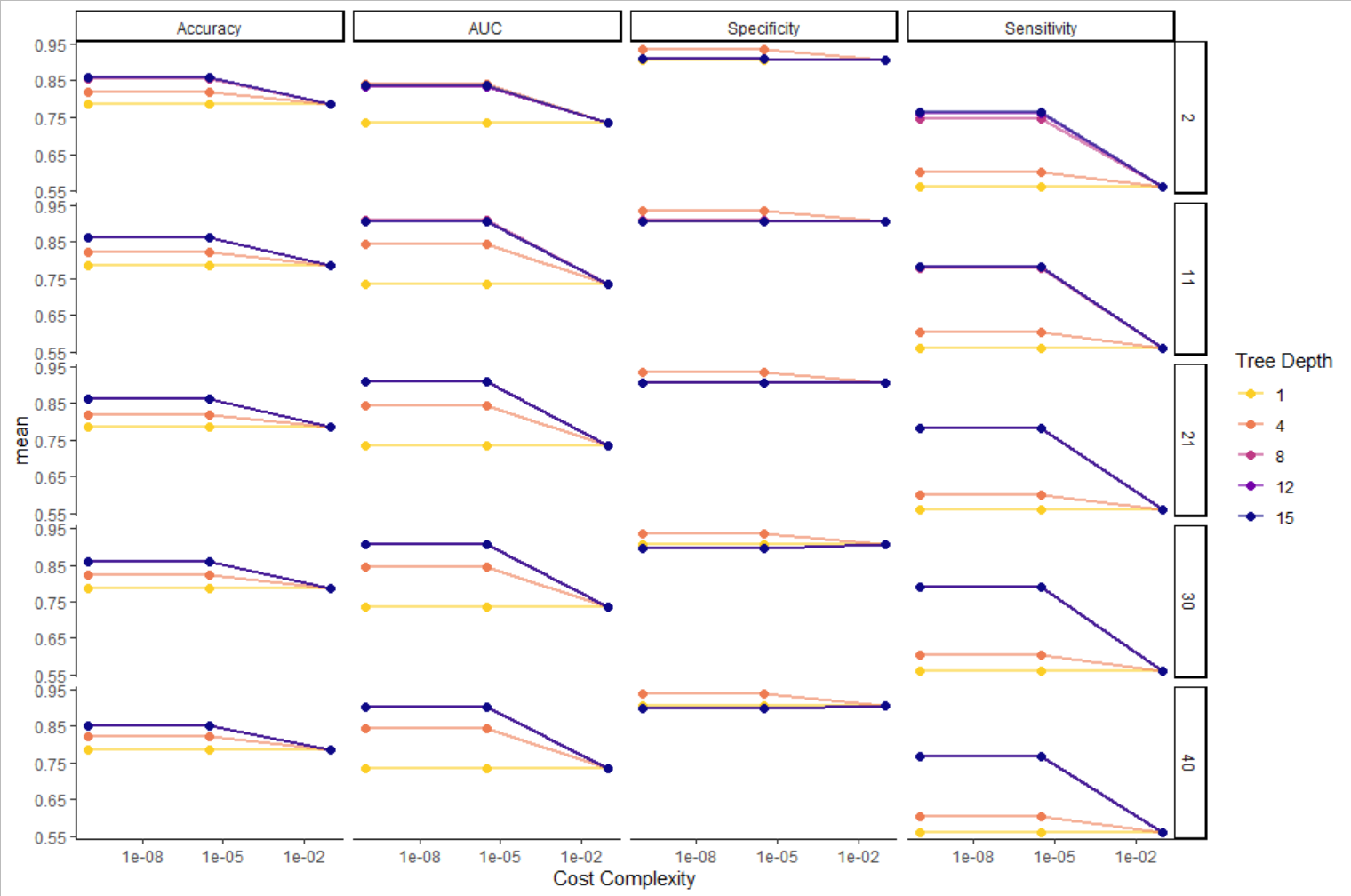


Supplemental Figure 3: Mean metrics for initial grid search by cost complexity, tree depth, and minimum split size for English self-administered version


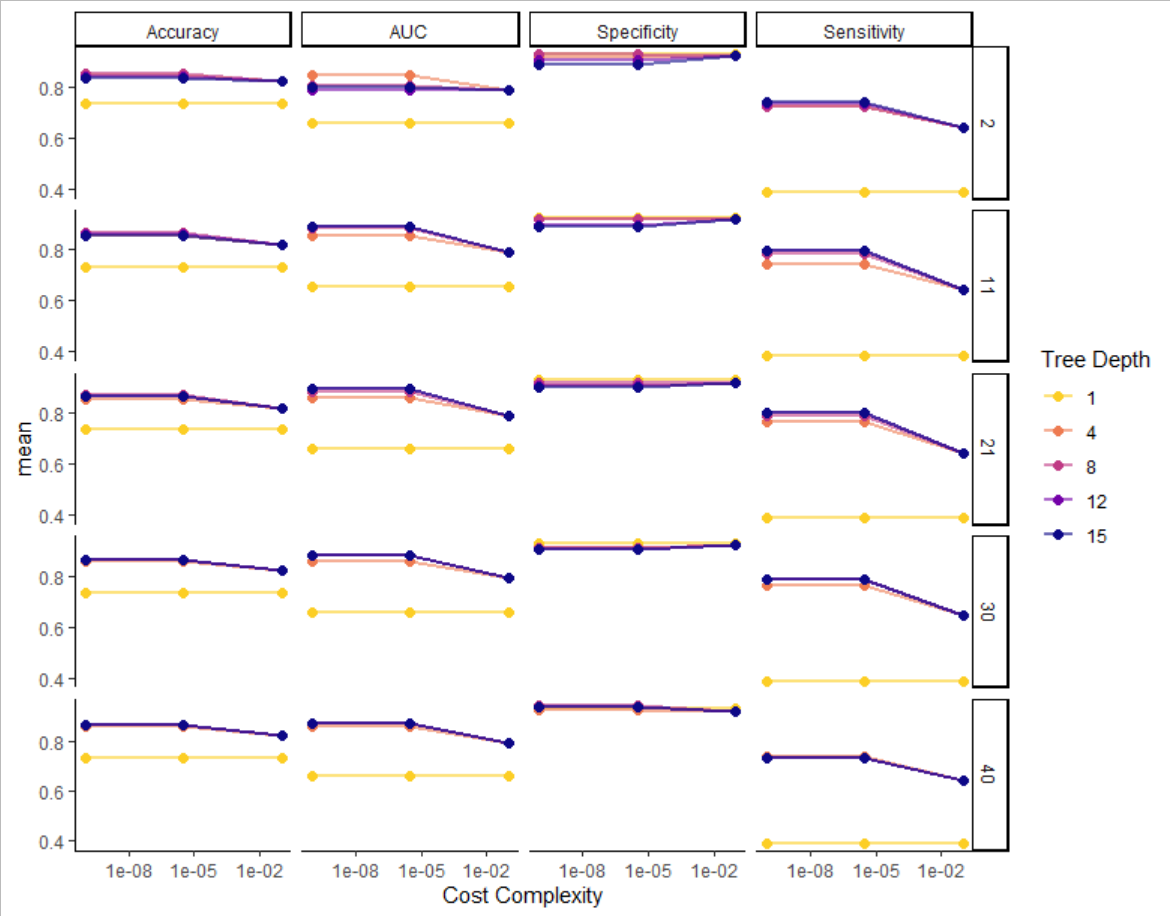


Supplemental Figure 4: Mean metrics for initial grid search by cost complexity, tree depth, and minimum split size for Spanish self-administered version


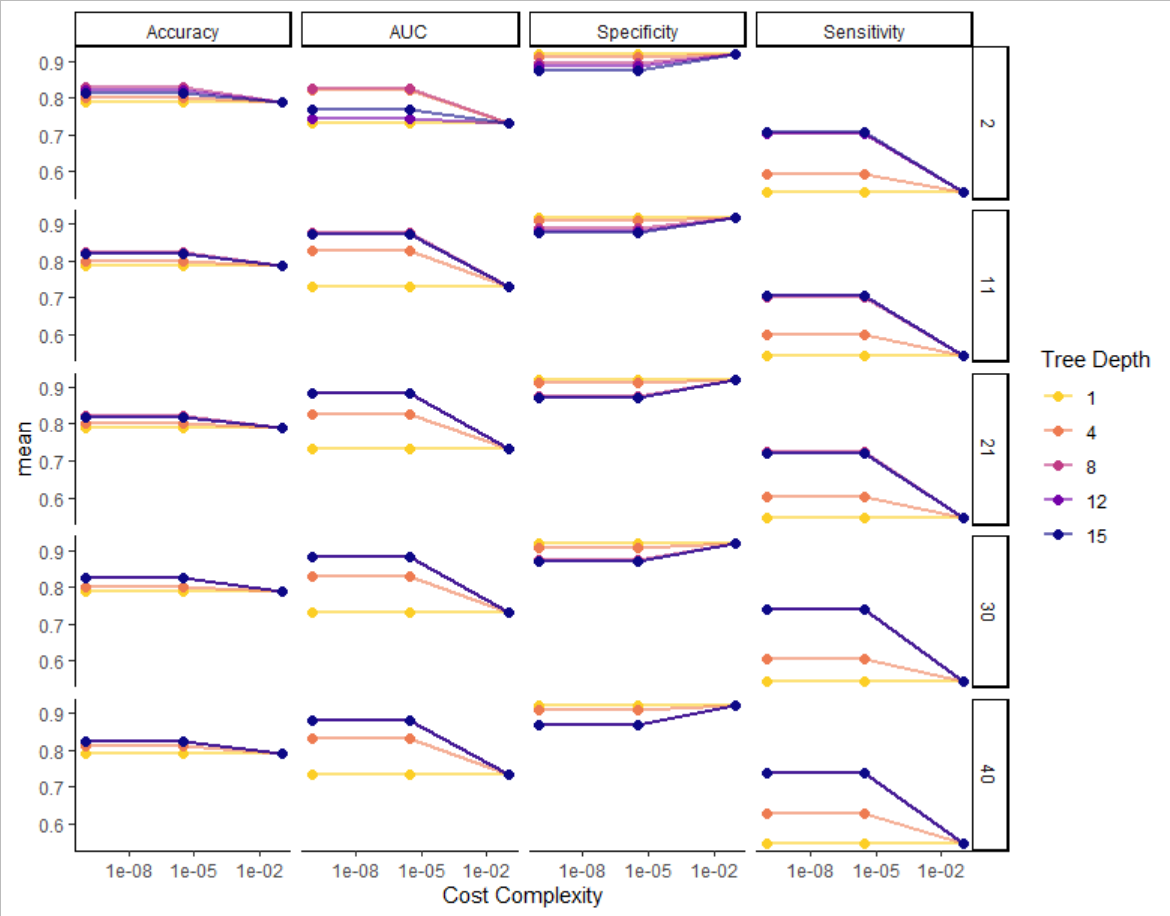


Supplemental Figure 5: Mean metrics for fine-tuned grid search by tree depth, penalty, and type of tree


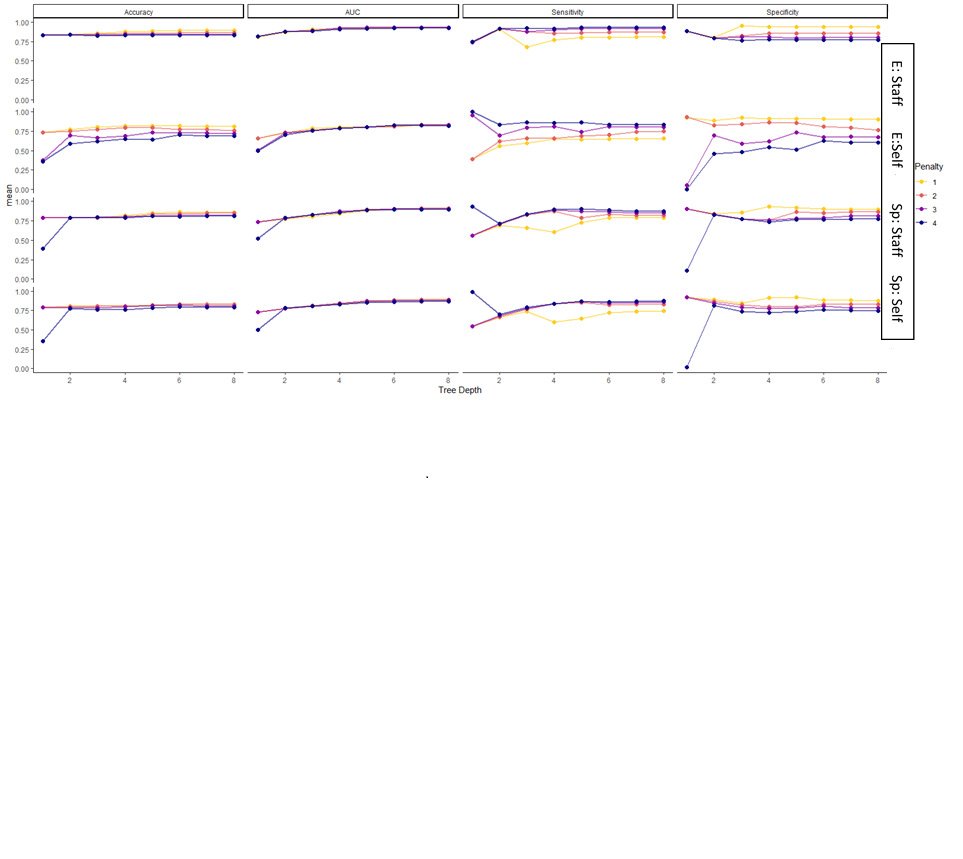


E: English, Sp: Spanish, Staff: Staff-administered, Self: Self-administered
